# Pharmacists’ perceptions of the Canadian opioid regulatory exemptions on patient care and opioid stewardship

**DOI:** 10.1177/17151635211034530

**Published:** 2021-08-16

**Authors:** Lisa D. Bishop, Zahava R. S. Rosenberg-Yunger, Shelita Dattani

**Affiliations:** School of Pharmacy, Memorial University of Newfoundland, St. John’s, Newfoundland; Ted Rogers School of Management, Health Services Management, Ryerson University, Toronto; Neighborhood Pharmacy Association of Canada, Toronto, Ontario

## Abstract

**Background:**

This study explored the perceptions of Canadian pharmacists about the barriers and facilitators of providing opioid stewardship activities in pharmacy practice, considering the subsection 56(1) class exemption under Health Canada’s Controlled Drugs and Substances Act (CDSA).

**Methods:**

Qualitative key informant telephone interviews were conducted with a convenience sample of pharmacists from across Canada. We included community or primary health care team-based pharmacists who self-identified as having experience with providing care for patients using opioids via the exemptions. All transcripts were de-identified, and thematic analysis was conducted to identify themes. Ethics approval was obtained.

**Results:**

Twenty pharmacists from community and primary health care teams, from all provinces and from urban and rural practices were interviewed. The following themes emerged: 1) optimization of opioid-related patient care, 2) jurisdictional impact and 3) awareness and education. Barriers and facilitators for opioid stewardship activities were identified.

**Discussion:**

The exemptions facilitated the pharmacists’ ability to provide opioid stewardship and positively affect patient care by providing continuity of and timely access to care. Our research demonstrated that pharmacists can responsibly and independently manage opioid prescriptions within this expanded scope, demonstrating the valuable contribution pharmacists can have in opioid stewardship.

**Conclusion:**

Pharmacists were willing and able to care for patients receiving opioid medication and thereby played a role in helping address the opioid crisis. The benefits of these exemptions were demonstrated beyond situations related to the COVID-19 pandemic and warrant consideration for consistent implementation across provincial and territorial jurisdictions, thereby ensuring equitable access to care for all Canadians.

**Knowledge Into Practice**

- The COVID-19 pandemic led to challenges for patients receiving opioids to receive continued care and thereby highlighted limitations in the pharmacists’ scope of practice in providing uninterrupted care.
- Our research provides evidence demonstrating the role pharmacists can play in appropriately and safely managing patients’ opioid therapy using the Controlled Drugs and Substances Act subsection 56(1) class exemption.
- Our study supports the role pharmacists can have in caring for patients using opioids, which warrants further consideration for permanent and consistent implementation of the exemptions to ensure equitable access to care.

**Mise En Pratique Des Connaissances**

- Avant la pandémie de la COVID-19, les pharmaciens avaient des difficultés à assurer la continuité des soins pour les patients utilisant des opioïdes en raison des limites de leur champ d’action.
- Notre recherche fournit des preuves qui démontrent le rôle que les pharmaciens peuvent jouer dans la gestion adéquate et sûre du traitement des patients par des opioïdes en utilisant l’exemption objective du paragraphe 56(1) de la Loi réglementant certaines drogues et autres substances.
- Notre étude soutient le rôle que les pharmaciens peuvent jouer dans la prise en charge des patients utilisant des opioïdes, ce qui ouvre la voie à l’application permanente et cohérente des exemptions afin de garantir un accès équitable aux soins.

## Introduction

During the COVID-19 pandemic, there were challenges in ensuring continuity of care for patients who were receiving opioids and controlled drugs. This was a notable concern for vulnerable populations, who may have experienced interrupted care due to barriers in accessing health care.^1^ Community pharmacies were considered an essential service during the pandemic and remained open in Canada and across the globe.^2^ There was a need for pharmacists to work to their full scope to help patients continue to receive care, including ongoing assessments and timely access to opioid medications. Prior to the pandemic, pharmacists were limited in their ability to provide this much-needed continuity of care.

Recognizing this gap, in March 2020, Health Canada issued a short-term subsection 56(1) class exemption under the Controlled Drugs and Substances Act (CDSA) and its Regulations permitting pharmacists to extend, transfer, receive verbal orders and deliver controlled substances.^3,4^ At the time of this study, the CDSA exemptions (hereafter referred to as “exemptions”) were temporary, expiring on September 30, 2021. The exemptions were recently extended for 5 years, ending on September 30, 2026. Some jurisdictions have also permitted pharmacists to adapt opioids and controlled substances (e.g., modify dosage, formulation, regimen, quantity), allowing them to support optimization of drug therapy regimens, including deprescribing as appropriate.^4^ The ability to adapt is not considered an exemption but is part of permanent regulations under the CDSA that permit pharmacists to provide opioids as long as the quantity dispensed does not exceed the amount originally authorized.^5^

As medication experts and health care professionals (HCPs) with ongoing patient relationships, pharmacists can play a significant role as opioid stewards.^6–11^ Opioid stewardship is described as “coordinated interventions designed to improve, monitor and evaluate the use of opioids in order to support and protect human health.”^12^ Given the potential harms associated with opioids, pharmacists are in a prime position to help ensure appropriate opioid use, given they are trained medication experts and are among the most accessible HCPs.^13,14^

Throughout the pandemic, the opioid crisis has continued to escalate in Canada, with 5148 apparent opioid-related deaths between April and December 2020, which was an 89% increase from the same time period in 2019 (2722 deaths).^15^ Pharmacists have demonstrated their role in addressing this crisis through various opioid stewardship activities, such as deprescribing, monitoring opioid use disorder, providing education and promoting harm-reduction strategies.^8,9,16-24^ With the exemptions, pharmacists are now better equipped to help address the opioid crisis by providing optimal care for patients who receive opioid medications.^25^

To better understand the impact of the exemptions on pharmacists’ ability to provide patient care, we set out to explore Canadian pharmacists’ experiences with using the exemptions in practice.

## Methods

Qualitative key informant telephone interviews were conducted by L.D.B. and Z.R.S.R.-Y. between October 2020 and January 2021 with Canadian community or primary health care, team-based pharmacists who used the exemptions in practice. Pharmacists were included if they 1) self-identified as having experiences with providing patient care with opioid medications using the exemptions in a community or primary health care setting, 2) were knowledgeable about appropriate prescribing practices for opioids and 3) were current on the evidence regarding patient care related to opioid medications. Participants were excluded if they were not licensed to practice pharmacy in Canada.

A convenience sample of Canadian pharmacists was recruited for the study. Participants were identified using online searches of pharmacists practising as opioid stewards and through the Canadian Pharmacists Association membership. Recruitment occurred through snowball sampling and posting notices on social media (LinkedIn, Facebook, Twitter) and pharmacy bulletins. Study information was emailed to participants, and informed consent was received prior to the interviews.

Interviews were semistructured and followed an interview guide (Appendix 1, available in the online version of the article), with demographic questions asked at the beginning of the interview. All interviews were digitally recorded, transcribed using Sonix transcription software,^26^ verified by a research assistant and sent to participants to review prior to analysis. Pseudonyms have been used for participants’ responses.

A thematic analysis of the de-identified transcripts was conducted, focusing on the barriers and facilitators of using the exemptions.^27^ The analysis was inductive and involved line-by-line coding of transcripts. Constant comparison was used to examine relationships between and across codes and categories. Interviewing continued until saturation of themes was attained and no new themes emerged. Two coders (L.D.B. and Z.R.S.R.-Y.) independently analyzed the first 3 transcripts. The coders created a codebook, and a list of all emerging codes was updated until no unique codes emerged. The codebook and findings were shared with the research team to triangulate the coders’ interpretations. Discrepancies were discussed until consensus was reached. The remaining transcripts were divided between 2 coders (L.D.B. and Z.R.S.R.-Y.). NVivo software was used to code, store and organize the data.^28^

Ethics approval was granted from the Ryerson University Research Ethics Board and Newfoundland and Labrador’s Health Research Ethics Authority.

## Results

Each interview was approximately 45 minutes in length. Twenty pharmacists working in a variety of practice settings were interviewed (Table 1). Pharmacists discussed both facilitators and challenges of using the exemptions. Table 2 gives the provincial/territorial differences permitting the use of the exemptions. (Appendix 2, an expanded version of Table 2, outlines examples of supporting quotes for each exemption.)

**Table 1:** Characteristics of participating pharmacists

Pharmacist characteristics	*n* = 20
Average age, y (range)	37, (28-48)
Pharmacy education*	
Bachelor’s	18
Master’s	2
Doctor of pharmacy	3
Currently enrolled in doctor of pharmacy program	3
Hospital pharmacy residency training	3
Other certificates/training (e.g., pain management)	2
Gender (female)	11
Position*	
Academia	1
Manager	4
Owner	5
Primary health care team	3
Staff pharmacist	7
Relief pharmacist	3
Location*,^†^	
British Columbia	4
Alberta	2
Saskatchewan	1
Manitoba	2
Ontario	4
Quebec	2
New Brunswick	3
Nova Scotia	1
Prince Edward Island	1
Newfoundland and Labrador	2
Years of practice as a pharmacist	
0-10	11
11-20	7
21-30	2
Practice setting*	
Banner	9
Chain/franchise	7
Independent	6
Primary care team	3
Population*	
Town or rural area with fewer than 10,000 people	6
City or town with more than 10,000 but fewer than 100,000 people	3
City with more than 100,000 but fewer than 1 million people	6
City with 1 million or more people	5

* Some participants were captured in more than 1 category.

† No participants from Northwest Territories, Nunavut, or Yukon.

**Table 2:** Controlled Drugs and Substances Act (CDSA) exemptions in practice

	Provincial differences^4^	
Exemption category	Permitted	Prohibited
Deliver medication	AB, BC, MB, NB, NL, NS, NT, ON, PE, QU, SK	YT, NU
Extend/renew prescription	AB, NB, NL, NS, ON, PE, QU, SK, YT, NT	BC, MB, NU
Transfer prescriptions to another pharmacist	AB, BC, NB, NL, NS, ON, PE, QU, SK, YT, NT	MB, NU
Receive a verbal order from a prescriber	AB, BC, NB, NS, ON, PE, QU, SK, YT, NT	MB, NL, NU
Adapt prescriptions* (i.e., modify the dosage, formulation, or regimen)	MB, NB, NS, ON, QU	BC, AB, SK, PE, NL, YT, NT, NU

* The ability to adapt an opioid or controlled substance is not an exemption; it is part of permanent regulations under the CDSA that permit pharmacists to sell or provide opioids as long as the quantity dispensed does not exceed the amount originally authorized.^5^ These may include adjusting the formulation/dosage form, adjusting the dose and regimen, deprescribing with a planned process for reducing or stopping opioids and part-filling, or dispensing a quantity less than the original amount.

Analysis of the transcripts resulted in 3 main themes: 1) optimization of opioid-related patient care, 2) jurisdictional impact and 3) awareness and education. Some quotes have been edited for clarity.

### Theme 1: Optimization of opioid-related patient care

The exemptions enabled pharmacists to provide enhanced patient care, with opportunities to expand their role. This theme was broken into the following subthemes: continuity of care and collaboration.

#### Continuity of care

Given the challenges some pharmacists experienced in contacting prescribers during the pandemic, the exemptions enabled pharmacists to provide uninterrupted care to patients: “Some patients have been finding it hard to see their doctor or reach their doctor, and that can compromise the patient care and the continuity ” (Rob).

Many pharmacists commented on how the exemptions facilitated stronger patient relationships. This was often emphasized in smaller settings: “No matter when they call, they’re getting the same 2 voices and seeing the same 2 faces” (Lee).

However, this was more of a challenge in larger settings. As Logan noted,

*I think a lot of that comes down to the more one-to-one relationship between a person and their primary care provider versus their pharmacy, which may be 1 of several that they use. And the pharmacist who is there, which may be 1 of several who works there, which makes it more difficult to kind of forge that personal relationship with patients.*

John spoke about previous scope limitations that impaired his ability to help patients who were in pain: “In the past, I wouldn’t be able to do anything pretty much short of sending them to the emergency.” With the exemptions, John explained how he now felt empowered to help his patients: “For all the changes in policy during COVID, I think the CDSA exemptions have been, at least from my point of view, by far the biggest, most impactful thing.”

#### Collaboration

The ability to use the exemptions had less impact on the pharmacists’ scope for those working in interdisciplinary team settings, given the collaborative relationships between physicians and pharmacists. However, the exemptions facilitated better collaborative care between prescribers and community pharmacists. As Jeal explained,

*The CDSA exemptions started the conversation going. . . . I feel like there’s a lot more collaboration, it’s just so much easier picking up the phone or texting . . . which is so much easier than before.*

This was also evident through the pharmacists’ ability to support prescribers during the pandemic.

*In the current pandemic situation, I think they’ve actually realized that it can be really nice for them to get to kind of relinquish control and say, ok, but I can let somebody else help me do my job.* (Susie Q)

Collaboration was fostered by sharing patient information across the health care team. One pharmacist explained that lack of patients’ medical history made it challenging to make decisions or recommendations and suggested that including indications on prescriptions would be helpful.

*Some physicians are resistant to reducing doses for patients. But to be fair, I don’t know what it’s like on their side . . . I don’t necessarily know what’s going on entirely with the patients. . . . It’s really hard to collaborate with physicians when you don’t have the whole picture . . . if they mandated a diagnosis on all of their prescriptions, then we would have a better idea. (David)*

Some pharmacists noted that prescribers were more open to collaboration during the pandemic and appreciated the pharmacists’ ability to assess their patients and extend prescriptions. This was especially useful for patients receiving opioid agonist therapy when the prescribers were unable to see their patients.

*I can still do my own assessment there to say whether it’s OK to continue to provide the dose until they can be seen by their physician. So, they feel a little bit more comfortable knowing that they’re not sort of blindly extending a prescription. There’s somebody that’s going to be sort of, I guess, following up with them or at least assessing somewhat until they have a chance to assess them themselves.* (Alex)

However, frustration was expressed in situations where the pharmacists were expected to extend opioid prescriptions when they could not reach the prescriber to discuss the prescription. Matthew discussed his concern that “opioid prescribing has always been a bit of a problem in our country,” and now he was “put in a position where I actually have to put my name on these prescriptions in order to extend them. It’s especially difficult when I can’t necessarily get hold of primary prescribers like I used to because they’ve very much cut back on their office hours.”

One pharmacist also mentioned that some physicians were resistant to pharmacists extending prescriptions, and this lack of collaboration was sometimes felt to be a barrier to providing care.

### Theme 2: Impact of regulations

The jurisdiction in which pharmacists practised directly affected their ability to perform opioid stewardship activities, given the differences in how the regulations were enacted provincially (Table 2).^4^ The exemptions expanded pharmacists’ scope but also affected day-to-day pharmacy operations, including their workflow and workload. This theme was divided into the following subthemes: operational and administrative considerations and use of and congruence in scope.

#### Operational and administrative considerations

The exemptions affected pharmacy operations, by both facilitating and hindering workflow. As Lance explained,

*Being able to accept orders by fax and accepting verbal orders just really helps streamline, rather than having . . . a lengthy back and forth whenever there is a discrepancy on a prescription*.

In some provinces, there is a regulatory requirement to write opioids on controlled prescription pads to help prevent forgeries and inappropriate prescribing. This resulted in workflow challenges with verbal orders. As Julie noted,

*We spent a not insignificant amount of time each week just attaching faxes to original triplicates, getting in touch with physicians to get the original triplicates that haven’t shown up.*

Given workflow and workload challenges, pharmacists were often faced with inadequate staff and insufficient time to spend with patients. As John explained,

*You could take the time for counselling, but we make these plans, and they don’t happen. And the biggest issue, I think that has to do with making or finding adequate staff and just kind of managing the pressure on prescriptions and other services like flu shots especially.*

Some pharmacists noted that other prescribers (e.g., dentists, nurse practitioners, physicians) were overburdened with work due to the pandemic, and the pharmacist was able to help fill the gap through their authority to manage opioid prescriptions. Logan explains how discrepancies in prescriptions were easier to rectify without needing to contact the prescriber. “And that is a very easy change to make now, which was not quite so easy in the past.”

#### Use of and congruence in scope

Many pharmacists expressed the desire to leverage their scope of practice as an integral member of the health care team through the use of the exemptions. As John explained,

*I think as pharmacists we’re probably able to use a lot more of our clinical knowledge in direct patient care. And . . . our hands aren’t tied by rules.*

Given the concern for patient safety with opioids, pharmacists demonstrated appropriate clinical judgement within their scope, “because you know that opioid medications can be abused” (Rob).

Timely and complete access to patient histories through an electronic health record (EHR) was noted as a way to help support more effective patient assessments and thereby help in making clinical decisions. This was particularly helpful as related to adapting and extending prescriptions.

*If I was worried clinically about a decision like their kidney function, being able to hop on [the EHR] and look up all that information makes a drastic change to practice.* (Jennifer)

Opportunities to further expand the pharmacists’ scope were noted by some pharmacists, especially those who were in jurisdictions that were not permitted to adapt prescriptions. Maria expressed a desire for the exemptions to be broader in scope,

*Why are we not able to have prescriptive authority for opioids and controlled medications for continuity of care? . . . I have no prescriptive authority to taper independently, I can’t modify prescription dosages, I can’t modify drug class . . . it’s just ridiculous*.

Given the inconsistencies in the legislative enactment of the exemptions across the provinces, some pharmacists thought the differences in scope of practice should be addressed to ensure equitable patient care. As Matthew noted, “I think that we could do good things with these exemptions as long as they are implemented appropriately and fairly across the board.” For example, David expressed his frustration when he was unable to use transfers or verbal orders and the negative impact it had on patient care,

*We can’t transfer to a pharmacy. . . . We can’t take verbal prescriptions . . . which would again make things a little easier because people don’t want to be doing interprovincial travel right now. . . . I wish we had adopted some of those [exemptions].*

Many pharmacists commented on how the exemptions enabled them to deliver expanded services. Lance discussed how an expanded remuneration model would facilitate the uptake of his expanded role as an opioid steward when using the exemptions: “We’re not going to have workplaces that facilitate us taking this role unless there is compensation for the employers.”

Most pharmacists were supportive of the exemptions becoming permanent. Many noted the opportunity for better communication, “more seamless care to patients” (Lee) and how the exemptions facilitated pharmacists using “a lot more of our clinical knowledge” (John). A few pharmacists discussed the need to be more nuanced in the implementation of the exemptions: “They could probably afford to have some more nuance in terms of when it is appropriate to apply them” (Logan).

### Theme 3: Awareness and education

Awareness and education were identified as a main theme and divided into the following subthemes: patient awareness and education, other HCPs’ awareness and education and pharmacists’ awareness and training.

#### Patient awareness and education

The exemptions facilitated the opportunity for increased patient awareness of the pharmacists’ role in managing opioid therapy, outside of their dispensing role: “It’s showing the patient that we do have that expertise and we do have that knowledge to be able to make that decision” (Jennifer).

Educating patients around the optimal use of opioids was also highlighted as important. As David noted, “It’s always easier to [educate] them at the beginning than for someone who’s been on opiates for however many years.” Focusing on pain management with patients that included nonpharmacologic options and opioid deprescribing could have a positive impact. As Lee noted,

*One thing that we recently launched was a pain management program that patients could enroll in which would involve a number of different touchpoints with myself as the pharmacist.*

Making patients aware of each HCP’s role in the circle of care was thought to be beneficial. As Rob explained,

*To educate the patient (both by the pharmacies and the doctor) that what the pharmacists can do and what as a doctor they can do, because everybody has their practice within the scope.*

#### Other HCPs’ awareness and education

Increasing the awareness of HCPs’ roles in the management of patients with chronic pain was noted as a way to help facilitate better patient care. Lee suggested that formalizing the expectations for collaboration would help,

*Having [interprofessional communication] required and funded, so that prescribers have some form of regular communication with the pharmacist and the rest of the team regarding patient status and progress would be helpful.*

Some pharmacists noted that all HCPs should be aware of current best practice guidelines when caring for patients receiving opioids. As Tracy noted, “Education is huge for all members of the health care team.” Barriers to the provision of optimal patient care were highlighted when team members had inconsistent practices, which was increasingly evident as pharmacists used the exemptions.

In provinces where electronic prescribing was used, it appeared to be a helpful system that saved time. However, Logan had concerns that sometimes “prescribers don’t know how to enter a prescription into the drug information system and how it is going to be viewed on the other end.” In these situations, he was able to use the exemptions to modify the prescription. Logan suggested that additional prescriber training would help rectify some of these issues so that e-prescribing could be a more efficient process and modifications to the prescriptions would not be necessary.

#### Pharmacists’ awareness and training

Most pharmacists were comfortable using the exemptions. However, some were unaware of the exemptions or lacked the confidence to manage opioid therapy. As Susan explained,

*I think that there’s still a lot of, you know, people out there who don’t necessarily realize what the changes are and what can be done. And then there’ll still be people who aren’t comfortable with doing it. (Susan)*

Given the dangers of opioids, Dawn suggested that mandatory training in opioid dispensing would help pharmacists to be more comfortable with providing care related to opioid medications: “I think that more education in general, not only just for safe supply and how we prescribe it, but mandatory, just like we have with injections.”

Support from colleagues through an informal practice support network was felt to be an important source of guidance. This was especially beneficial in smaller settings. As Jennifer discussed,

*When you’re working alone and you don’t know the answer or something, having that network of people to check in with and say, what do you think about this, and being supportive of each other is a great help.*

It was suggested by some that pharmacists could play a larger role in opioid stewardship through the assessment, treatment and management of chronic pain and opioid deprescribing. As Maria stated, “Education plays a big role in opioid stewardship,” and she suggested that more education for pharmacists and pharmacy students would help support their role.

## Discussion

Throughout our study, pharmacists indicated that the exemptions have facilitated their ability to provide opioid stewardship and improve patient care. Our research demonstrated that pharmacists can responsibly and independently manage opioid prescriptions within the expanded scope provided by the exemptions. The exemptions saved time for patients by decreasing delays in access to their medications and providing continuity of care. Pharmacists most commonly reported using verbal orders, deliveries and transfers, as these were easier to incorporate into their workflow. Although extensions and adaptations were used less commonly, many of the pharmacists in our study were willing to use this expanded scope, if given the opportunity, and were receptive to further expansions into initiating opioid prescriptions in the context of deprescribing and opioid agonist therapy.

As an integral member of the health care team, pharmacists can more fully contribute to the patients’ circle of care. Good collaboration and communication between HCPs was expressed by pharmacists in our study as a way to facilitate continuity of care. For those working in interdisciplinary team settings, the prescribers were generally more accessible, and therefore, pharmacists were able to develop good collaborative relationships, which enabled stewardship activities.^29,30^ In addition, community pharmacists who worked in rural or smaller pharmacies tended to have closer relationships with prescribers, which also facilitated patient care.

Increasing awareness of HCPs about each other’s roles as well as more pharmacist training in using the exemptions were suggested as ways to help increase the pharmacists’ confidence in the optimal use of the exemptions and thereby increase stewardship activities.^31–33^ Access to patient information (e.g., EHR) was also expressed as a way to facilitate opioid stewardship, as this would enable more thorough patient assessment when using the exemptions.^34,35^ Improved access to nonpharmacologic options for patients may also lead to greater success with pain management and opioid deprescribing.^14,36,37^

Our study highlighted some system inconsistencies resulting in inequalities related to patients’ ability to access care. Barriers to the provision of expanded scope activities resulted from inconsistent access to resources (e.g., time, staffing, workload), and it was suggested that appropriate remuneration would help provide more resources to support these activities. This is supported by the economic value that pharmacists can provide to the health care system.^29,38–40^ In addition, in provinces where some of the exemptions were prohibited, there were noted barriers to patient care. Policy harmonization with respect to consistent enactment of the exemptions across provinces/territories would help ensure more equitable access to care across jurisdictions.

There were several limitations to this research. Although there was representation from all 10 provinces, nobody participated from the territories, and the views of participants may not be representative of all pharmacists from across the country. Given the provincial differences in how the exemptions were enacted and the varied scope of practice, the findings may not have captured the nuances within each jurisdiction. Since only pharmacists who implemented the exemptions were interviewed, this research does not represent the opinions of all pharmacists or capture the full extent of implementation. This research focused on the pharmacist perspective; however, to fully understand the impact of the exemptions, perspectives from other prescribers and patients would be helpful.

Future research should explore how the exemptions have been implemented provincially and their subsequent impact on patient care and the pharmacists’ scope of practice. Further research into the economic value of pharmacists providing expanded-scope activities related to the exemptions would provide evidence for an appropriate remuneration model and sustainability of these activities. Exploration into the ability to provide the full scope of services in rural vs urban settings would also be helpful, as would an investigation into how collaboration can be enhanced in community pharmacy settings. Further research is needed to determine how the pharmacists’ scope could be expanded by initiating opioid therapy. The positive impact that pharmacists had in caring for patients with opioid use disorder was evident through this research and will be explored further in a separate publication.

Pharmacists in our study were willing and comfortable to expand their role in providing patient care by using the exemptions. We recommend the following policy modifications:

1. Provinces/territories should authorize pharmacists to fully utilize the exemptions and adaptation ability to support the unique challenges within the different jurisdictions. For example, British Columbia cannot extend or adapt; Manitoba cannot extend, transfer or accept verbal orders; and Newfoundland and Labrador cannot adapt or accept verbal orders; however, Ontario, Quebec, New Brunswick and Nova Scotia are able to use the full range of abilities.^4^
2. While further research is necessary to fully understand the impact of this expanded role, our study highlighted, from the pharmacist’s perspective, the need to make the exemptions permanent.^41^

## Conclusion

Pharmacists shared their experiences with using the exemptions in many aspects of their practice. Our research provided evidence demonstrating the role pharmacists can play in appropriately and safely managing patients’ opioid therapy and contributing to opioid stewardship. These exemptions have enabled pharmacists to have a positive impact on patient care and have facilitated the continuity of care during a time when patients required timely access to services. The 5-year extension of the exemptions has provided an opportunity for pharmacists to continue to provide this uninterrupted care. The benefits of these exemptions were demonstrated beyond issues related to the COVID-19 pandemic and warrant consideration for consistent implementation across provincial and territorial jurisdictions.^41^ This will help close the gap across jurisdictions and ensure universal and equitable access to care for all Canadians. ■

## Supplemental Material

sj-pdf-1-cph-10.1177_17151635211034530 – Supplemental material for Pharmacists’ perceptions of the Canadian opioid regulatory exemptions on patient care and opioid stewardship Click here for additional data file. Supplemental material, sj-pdf-1-cph-10.1177_17151635211034530 for Pharmacists’ perceptions of the Canadian opioid regulatory exemptions on patient care and opioid stewardship by Lisa D. Bishop, Zahava R. S. Rosenberg-Yunger and Shelita Dattani in Canadian Pharmacists Journal / Revue des Pharmaciens du Canada

sj-pdf-2-cph-10.1177_17151635211034530 – Supplemental material for Pharmacists’ perceptions of the Canadian opioid regulatory exemptions on patient care and opioid stewardship Click here for additional data file. Supplemental material, sj-pdf-2-cph-10.1177_17151635211034530 for Pharmacists’ perceptions of the Canadian opioid regulatory exemptions on patient care and opioid stewardship by Lisa D. Bishop, Zahava R. S. Rosenberg-Yunger and Shelita Dattani in Canadian Pharmacists Journal / Revue des Pharmaciens du Canada

## References

[1] McMahon M Nadigel J Thompson E Glazier RH. Informing Canada’s health system response to COVID-19: priorities for health services and policy research. Healthc Policy 2020;16(1):112-24. 10.12927/hcpol.2020.26249 PMC7435075 32813643

[2] Gregory PAM Austin Z. COVID-19: how did community pharmacies get through the first wave? Can Pharm J 2020;153(5):243-51. 10.1177/1715163520945741 PMC7429913 33106756

[3] Health Canada. Exemptions for practitioners and pharmacists prescribing and providing controlled substances, and for patients, during the coronavirus pandemic. 2020. Available: https://www.canada.ca/en/health-canada/services/health-concerns/controlled-substances-precursor-chemicals/policy-regulations/policy-documents/section-56-1-class-exemption-patients-pharmacists-practitioners-controlled-substances-covid-19-pandemic.html (accessed Mar. 19, 2021).

[4] Canadian Pharmacists Association. COVID-19 and controlled drugs and substances.; 2020. Available: https://www.pharmacists.ca/cpha-ca/assets/File/cpha-on-the-issues/CovidCDSA.pdf (accessed Jun. 30, 2021).

[5] Health Canada. Prescription management by pharmacists with controlled substances under the Controlled Drugs and Substances Act and its regulations. 2020. Available: https://www.canada.ca/en/health-canada/services/health-concerns/controlled-substances-precursor-chemicals/policy-regulations/policy-documents/prescription_management_pharmacists_controlled_substances.html (accessed Mar. 31, 2021).

[6] Murphy L Chang F Dattani S Sproule B. A pharmacist framework for implementation of the Canadian Guideline for Opioids for Chronic Non-Cancer Pain. Can Pharm J 2019;152(1):35-44. 10.1177/1715163518818200 PMC6346336 30719196

[7] Bhimji H Landry E Jorgenson D. Impact of pharmacist-led medication assessments on opioid utilization. Can Pharm J 2020;153(3):148-52. 10.1177/1715163520908285 PMC7265577 32528598

[8] Compton WM Jones CM Stein JB Wargo EM. Promising roles for pharmacists in addressing the U.S. opioid crisis. Res Soc Admin Pharm 2019;15(8): 910-6. 10.1016/j.sapharm.2017.12.009 PMC7373345 29325708

[9] Reynolds V Causey H McKee J Reinstein V Muzyk A. The role of pharmacists in the opioid epidemic: an examination of pharmacist-focused initiatives across the United States and North Carolina. North Carolina Med J 2017;78(3):202-5. 10.18043/ncm.78.3.202 28576963

[10] Barry AR Chris CE. Treatment of chronic noncancer pain in patients on opioid therapy in primary care: a retrospective cohort study. Can Pharm J 2020;153(1):52-8. 10.1177/1715163519887766 PMC6966273 32002103

[11] Iqbal A David Knaggs R Anderson C Toh LS. Role of pharmacists in optimising opioid therapy for chronic non-malignant pain; a systematic review [published online Nov. 24, 2020]. Res Social Adm Pharm. 10.1016/j.sapharm.2020.11.014 33309322

[12] ISMP Canada. Opioid stewardship. Available: https://www.ismp-canada.org/opioid_stewardship/ (accessed Feb. 22, 2021).

[13] Tsuyuki RT Beahm NP Okada H Al Hamarneh YN. Pharmacists as accessible primary health care providers: review of the evidence. Can Pharm J (Ott) 2018;151(1):4-5. 29317929 10.1177/1715163517745517 PMC5755826

[14] Busse J. The 2017 Canadian guideline for opioids for chronic non-cancer pain. Cancer Pain 2017;105.

[15] Government of Canada. Opioid-related harms in Canada. Available: https://health-infobase.canada.ca/substance-related-harms/opioids/ (accessed Jul. 1, 2021).

[16] Rosenberg-Yunger ZRS Ellen M Mickleborough TMe . The North American opioid experience and the role of community pharmacy. J Public Health Manage 2018;24(4):301-5. 10.1097/PHH.0000000000000807 29787504

[17] Brown G. Opioid stewardship: moving beyond the “why” to the “how.” Can J Hosp Pharm 2020;73(1):3-4. 32109953 PMC7023919

[18] Murphy L Babaei-Rad R Buna D , et al. Guidance on opioid tapering in the context of chronic pain: evidence, practical advice and frequently asked questions. Can Pharm J 2018;151(2):114-20. 10.1177/1715163518754918 PMC5843113 29531629

[19] Suddaby RJ Morris CJ Gray B. Role of New Zealand community pharmacists in opioid substitution treatment. Res Soc Admin Pharm 2019;15(5):e20-1.

[20] Bach P Hartung D. Leveraging the role of community pharmacists in the prevention, surveillance, and treatment of opioid use disorders. Addict Sci Clin Pract. 2019;14(1):30. 31474225 10.1186/s13722-019-0158-0 PMC6717996

[21] Bratberg JP Kubicsko D. Meeting people where they’re at: a focus on pharmacist harm reduction roles in the opioid crisis. J Am Coll Clin Pharm 2020;3(2):400-3.

[22] Wertheimer AI Lai L. The pharmacists’ contribution against opioid addiction. J Pharm Health Serv Res 2020;11(3):231-5.

[23] Bailey AM Wermeling DP. Naloxone for opioid overdose prevention: pharmacists’ role in community-based practice settings. Ann Pharmacother 2014;48(5):601-6. 10.1177/1060028014523730 PMC4361936 24523396

[24] Tsuyuki RT Arora V Barnes M , et al. Canadian national consensus guidelines for naloxone prescribing by pharmacists. Can Pharm J 2020;153(6):347-51. 10.1177/1715163520949973 PMC7689616 33282024

[25] Canadian Pharmacists Association. CPhA opioid action plan. Available: https://www.pharmacists.ca/cpha-ca/assets/File/cpha-on-the-issues/CPhA_OpioidActionPlan-18Nov16.pdf (accessed Oct. 21, 2020).

[26] Sonix. Available: https://sonix.ai/ (accessed Mar. 19, 2021).

[27] Densin N Lincoln Y. The SAGE handbook of qualitative research. Thousand Oaks (CA): SAGE; 2021.

[28] NVivo. Qualitative data analysis software. Available: https://www.qsrinternational.com/nvivo-qualitative-data-analysis-software/home (accessed Mar. 19, 2021).

[29] Raiche T Pammett R Dattani S , et al. Community pharmacists’ evolving role in Canadian primary health care: a vision of harmonization in a patchwork system. Pharm Pract (Granada) 2020;18(4).:2171. 10.18549/PharmPract.2020.4.2171 PMC7603659 33149795

[30] Khaira M Mathers A Benny Gerard N Dolovich L. The evolving role and impact of integrating pharmacists into primary care teams: experience from Ontario, Canada. Pharmacy 2020;8(4):234. 10.3390/pharmacy8040234 PMC7768418 33297509

[31] Necyk C Cor K Mazzuca A Meleshko L. An evaluation of Alberta pharmacists’ practices, views and confidence regarding prescription drug abuse and addiction within their practice setting. Can Pharm J 2019;152(6):376-87. 10.1177/1715163519865914 PMC6851641 31762848

[32] Hussein R Whaley CRJ Lin ECJ Grindrod K. Identifying barriers, facilitators and behaviour change techniques to the adoption of the full scope of pharmacy practice among pharmacy professionals: using the theoretical domains framework. Res Soc Admin Pharm 2020;17(8):1396-1406. 10.1016/j.sapharm.2020.10.003 34165083

[33] Gallagher RM Gallagher HC. Improving the working relationship between doctors and pharmacists: is inter-professional education the answer? Adv Health Sci Educ Theory Pract 2012;17(2):247-57. 10.1007/s10459-010-9260-5 21088991

[34] Leong C Alessi-Severini S Sareen J Enns MW Bolton J. Community pharmacists’ perspectives on dispensing medications with the potential for misuse, diversion, and intentional overdose: results of a province-wide survey of community pharmacists in Canada. Subst Use Misuse 2016;51(13):1724-30. 10.1080/10826084.2016.1197261 27487323

[35] Canada Health Infoway. Connected health information in Canada: a benefits evaluation study. Available: https://www.infoway-inforoute.ca/en/component/edocman/resources/reports/benefits-evaluation/3510-connected-health-information-in-canada-a-benefits-evaluation-study-document (accessed Mar. 19, 2021).

[36] Vitoula K Venneri A Varrassi G , et al. Behavioral therapy approaches for the management of low back pain: an up-to-date systematic review. Pain Ther 2018;7(1):1-12. 29767395 10.1007/s40122-018-0099-4 PMC5993685

[37] Chang K-L Fillingim R Hurley RW Schmidt S. Chronic pain management: nonpharmacological therapies for chronic pain. FP Essent 2015;432:21-6. 25970869

[38] Marra C Johnston K Santschi V Tsuyuki RT. Cost-effectiveness of pharmacist care for managing hypertension in Canada. Can Pharm J (Ott) 2017;150(3):184-97. 10.1177/1715163517701109 PMC5415065 28507654

[39] Sanyal C Husereau DR Beahm NP Smyth D Tsuyuki RT. Cost-effectiveness and budget impact of the management of uncomplicated urinary tract infection by community pharmacists. BMC Health Serv Res 2019;19(1):499. 31319844 10.1186/s12913-019-4303-y PMC6639967

[40] Gagnon-Arpin I Dobrescu A Sutherland G Stonebridge C Dinh T. The value of expanded pharmacy services in Canada. The Conference Board of Canada. Available: https://www.conferenceboard.ca/e-library/abstract.aspx?did=8721 (accessed Apr. 16, 2021).

[41] Rosenberg-Yunger ZR Bishop LD Dattani S . Modernizing Canadian pharmacists’ scope of practice for controlled drugs and substances. Canadian Health Policy. 2021. Available: https://www.canadianhealthpolicy.com/products/modernizing-canadian-pharmacists—scope-of-practice-for-controlled-drugs-and-substances.html (Jun. 10, 2021).

