# Supplementary material for: Pharmacists’ perceptions of the Canadian opioid regulatory exemptions on patient care and opioid stewardship
Source: Can Pharm J (Ott). 2021 Aug 16;154(6):394–403. doi: 10.1177/17151635211034530 (PMC8581809; doi:10.1177/17151635211034530)
Supplement: sj-pdf-1-cph-10.1177_17151635211034530 – Supplemental material for Pharmacists’ perceptions of the Canadian opioid regulatory exemptions on patient care and opioid stewardship [file sj-pdf-1-cph-10.1177_17151635211034530.pdf]

APPENDIX 1 Interview guide

Semi-structured interview questions
1. In terms of scope of practice, what are you able to do now given the CDSA exemptions that was different from before? Please explain any changes in scope which have affected your ability to provide patient care.
2. Please tell me about your experiences with patient care related to opioid medications. How haVE your experiences changed with the new CDSA exemptions?<br>**a.** Can you describe examples of how you've been able to use the exemptions in your practice?
3. Please describe your experiences in collaborating with prescribers regarding patient care related to opioid medications. How has this changed with the new CDSA exemptions?<br>a. Can you elaborate on the types of encounters you've had with prescribers?<br>b. How have your encounters changed with the CDSA exemptions?
4. Please describe your experiences when providing patients with care regarding their opioid prescriptions. How has this changed since the CDSA exemptions?<br>a. Can you elaborate on the ways that you have been able to help patients?<br>b. How have your patients responded to your ability to provide these services?
5. How have the CDSA exemptions affected your practice and your ability to provide care?<br>a. Describe situations where exemptions facilitated or hindered your ability to manage opioid prescriptions
6. What would be the impact on pharmacy practice and patient care if the CDSA exemptions became permanent?<br>a. Can you elaborate on the aspects of your practice or patient care that would be impacted?
7. Thinking beyond the CDSA exemptions, what are some of the barriers and facilitators with pharmacists being able to provide patient care related to opioid medications?<br>a. Describe situations where pharmacists <u>are able</u> to provide opioid stewardship activities<br>b. Describe situations where pharmacists <u>are not able</u> to provide opioid stewardship
8. What are the gaps, beyond the current regulations, that you think should be addressed in order to facilitate and increase uptake of opioid stewardship and improve patient care in pharmacy practice?<br>a. What are gaps related to collaborating with physicians?<br>b. What are gaps related to providing patient care?<br>c. What are the gaps related to your practice setting?
9. Is there anything you'd like to add about your practice and your ability to provide opioid stewardship?

Bishop LD, et al. Pharmacists' perceptions of the Canadian opioid regulatory exemptions on patient care and opioid stewardship. *Can Pharm J (Ott)* 2021;154. DOI: 10.1177/17151635211034530.
