# Supplementary material for: Pharmacists’ perceptions of the Canadian opioid regulatory exemptions on patient care and opioid stewardship
Source: Can Pharm J (Ott). 2021 Aug 16;154(6):394–403. doi: 10.1177/17151635211034530 (PMC8581809; doi:10.1177/17151635211034530)
Supplement: sj-pdf-2-cph-10.1177_17151635211034530 – Supplemental material for Pharmacists’ perceptions of the Canadian opioid regulatory exemptions on patient care and opioid stewardship [file sj-pdf-2-cph-10.1177_17151635211034530.pdf]

APPENDIX 2 CDSA exemptions in practice

Exemption category	Descriptive quotes	Provincial differences[4]	
		Permitted	Prohibited
Deliver medication	<u>Supportive quotes</u><br>*"Delivery was really helpful also with COVID because we have a lot of patients that can't go out."* [Mary]<br><br>*"Better care for that population, for everything from, you know, delivering of just any narcotic, a patient has a hip surgery and is at home and needs narcotics, to blister-pack patients. Just to be able to offer delivery by a non-pharmacist is quite big for us in our practice."* [Julie]	AB, BC, MB, NB, NL, NS, NT, ON,PE, QU, SK	YT, NU
Extend/ renew prescription	<u>Supportive quotes</u><br>*"Being in a very rural area, we're often the only service that is available to patients, especially on a walk-in basis… So rather than having to send her to either 811 or to emergency, like those are situations that I really feel are really appropriate for us to step in and extend prescriptions."* [Jennifer]<br><br>*"If we couldn't get a prescription for a controlled substance or narcotic for a resident of our personal care home, rather than having to contact the home staff and tell them that we need to prescribe for someone's medication because they're out of refills we just sort of reduce that pressure on the home staff to have to go and arrange for a refill."* [Alex]<br><br><u>Identification of a barrier</u><br>*"So as far as extensions and emergency dispenses are concerned, most pharmacists that I know, and especially those in the store that I'm working in ... we're really, really uncomfortable... we have access to the physician's cell phone and they're home, way more now."* [Dawn]	AB, NB, NL, NS, ON, PE, QU, SK, YT, NT	BC, MB, NU
Transfer prescriptions to another pharmacist	<u>Supportive quotes</u><br>*"Transferring is also super valuable because serving clients who often have to travel out of their own communities to access care away from home… And so being able to transfer their prescriptions rather than arrange for a new prescription to be sent to a pharmacy in their temporary destination, it's been a tremendous help."* [Lance]	AB, BC, NB, NL, NS, ON, PE, QU, SK, YT, NT	MB, NU

	"I don't think there's really a concern anymore about transferring narcotic prescriptions or having to worry about the legitimacy of them and stuff like that because any pharmacist can go on [the EHR] and just look up what people have been getting to make sure that it's accurate." [Bob]<br><br>Identification of a barrier<br>*"I think people are disregarding what would have been done in the past, which is to have 2 standing prescriptions at 2 separate stores, specifying dates, and just going straight to 'oh, they can be transferred all around, so it doesn't matter'. Which I feel like a mix of those 2 options would be best to serve the patient, both in terms of effectiveness and safety, which is my big concern with all that transferring."* [Logan]<br><br>"*Being able to transfer prescriptions would be helpful… for example, I have many patients that live on reserves, so they have to drive in every month to get the prescription… the reserves are really being hit by the pandemic and there are pharmacies close by… And I say, 'no, unfortunately, I can't transfer your Percocet'."* [David]		
Receive a verbal order from a prescriber	Supportive quotes<br>*"Just being able to get in touch with a prescriber and take verbal prescriptions. It is helpful for, you know, like weekends, dental prescriptions, late-night or weekend dental prescriptions for the narcotics."* [Julie]<br><br>"*Being able to accept orders by fax and accepting verbal orders just really helps streamline, rather than having … a lengthy back and forth whenever there is a discrepancy on a prescription. It's nice to just pick up the phone and contact the prescriber and get it sorted out immediately.*" [Lance]<br><br>Identification of a barrier<br>*"I fear that a lot less documentation is going on in the office, that physicians are simply just picking up the phone. There's a lot less patient interaction going on. So, I'm actually not a fan whatsoever of these new verbal order permissions." [Mathew]*<br><br>*"And it almost feels to me that it's not trusting of our abilities when [the college] makes rules like this, that we can't take a verbal order or something. It's almost like they're distrusting in our capability to take that order appropriately."* [Jennifer]	AB, BC, NB, NS, ON, PE, QU, SK, YT, NT	MB, NL, NU

	"I think that we should just be able to take a verbal order and then not necessarily have to follow up with the triplicate... it helps for sure to be able to do the verbal order, but it doesn't necessarily reduce that administrative and paper burden that we're still living in." [Mika]		
Adapt prescriptions* (i.e., modify the dosage, formulation, or regimen)	<u>Supportive quotes</u><br>*"Modifying the prescription quantity. When I do that, I think it's really optimizing care and it's reducing risk of diversion in the community."* [Maria]<br><br>*"[If] you get a "script" from the emergency room or from the surgeon and it's like Friday night at 6:00 and you're not able to join the doctor and you want to change the medication, to lower the dosage … Before [CDSA exemptions], you were not able to do that … I think this is the big change, is being able to adjust the therapy for the patient."* [Carlos]<br><br>*"We are now able to titrate down the medication… we have to respect the maximum dosage that was prescribed by the doctor, but we can lower the dose as we need to, to ensure security to the patient or to avoid side effects or things like that."* [Carlos]<br><br><u>Identification of a barrier</u><br>*"That is something I'm a little bit cautious about, given that I am currently not a permanent employee of any of these pharmacies that I'm working at. I don't want to set the expectation with clients that this is the new normal when I know that not all of my colleagues alongside me may-be comfortable with that."* [Lance]	MB, NB, NS, ON, QU	BC, AB, SK, PE, NL, YT, NT, NU

* The ability to adapt an opioid or controlled substance is not an exemption, it is part of permanent regulations under the CDSA that permit pharmacists to sell or provide opioids as long as the quantity dispensed does not exceed the amount originally authorized.[5] These may include adjusting the formulation/dosage form, adjusting the dose and regimen, deprescribing with a planned process for reducing or stopping opioids, and part-filling, or dispensing a quantity less than the original amount

Bishop LD, et al. Pharmacists' perceptions of the Canadian opioid regulatory exemptions on patient care and opioid stewardship. Can Pharm J (Ott) 2021;154. DOI: 10.1177/17151635211034530.
